# Study protocol: Apps and peer support for a healthy future and living well with diabetes (APHLID-M)

**DOI:** 10.1016/j.conctc.2025.101484

**Published:** 2025-04-14

**Authors:** K.O. Mathews, F. MacMillan, V. Wong, M. Craig, J.R. Greenfield, R. Hicks, T. Jones, A. Poynten, T. Wong, M. Reyes, K. Tannous, C. Wilson, P. Hay, S. Abdo, M.K. Piya, J. Lai, M. Venigalla, R. Thomson, D. Simmons

**Affiliations:** a School of Medicine, Western Sydney University, Campbelltown, NSW, Australia; b Office of the Senior Deputy Vice-Chancellor and Vice-President (Research, Enterprise and Global), Western Sydney University, Penrith, Australia; c Liverpool Hospital, Australia; d Sydney Children's Hospital Westmead, University of NSW, Australia; e St Vincent's Hospital, University of NSW, Australia; f Goulburn Valley Health, Australia; g Prince of Wales Hospital, Australia; h Bankstown Hospital, Australia; i St George Hospital, Australia; j Macarthur Diabetes, Endocrinology and Metabolism Service, Campbelltown Hospital, Campbelltown, NSW, Australia; k Analytical Edge Pty Ltd, Australia

**Keywords:** Diabetes, Mental, Health, Young adults, Digital health app

## Abstract

**Background:**

Mental health conditions are common among non-pregnant young people with any form of diabetes, affecting diabetes self-management and increasing complications risk. Limited evidence exists on whether smartphone applications “apps” combining diabetes and mental health (MH) support can improve self-management and MH in these young people. The Apps and Peer support for a Healthy future and Living Well with Diabetes (APHLID-M) multicentre study includes two randomised controlled trials (RCTs) testing such an app, aimed at reducing distress among young adults with diabetes with and without a mental health condition (MHC).

**Methods and analysis:**

An app containing diabetes and MH resources was configured onto a pre-existing, digital health platform. Young adults aged 16–30 years with diabetes will be recruited from eight Australian outpatient clinics, screened using the Kessler Psychological Distress Scale (K10) and the Problem Areas in Diabetes (PAID) questionnaires. Based on MH status, participants will be allocated to the primary RCT (MHC group) or a nested-exploratory RCT (No-MHC group) and randomised by site to the “app” (Intervention) or “no app” (control). All participants will have access to peer support and will continue to receive standard diabetes care through their clinic. Recruitment will end once 142 participants are enrolled in the primary RCT. The primary outcome is change in psychological distress (K10), and the secondary outcome change in HbA1c, assessed at baseline and 6 months.

**Discussion:**

APHLID-M will offer valuable insights into effects of digital technology in enhancing MH (particularly distress) physical health and well-being in young people with diabetes.

## 1 Background

Diabetes is a growing issue among young Australians, with >30,000 young adults aged 10–30 years registered with the National Diabetes Services Scheme, 78 % of whom have Type 1 diabetes mellitus (T1DM) [1]. Standardised mortality rates as high as 6.91 (95 % CI 5.56–8.59) and 4.59 (95 % CI 3.00–7.04) have been reported for females aged 20–29 with T1DM and type 2 diabetes mellitus (T2DM) respectively, while standardised mortality rates in similarly aged males were 2.95 (95 % CI 2.41–3.61) for T1DM and 4.00 (95 % CI 2.66–6.02) for T2DM [2]. Hospitalisation rates for severe hypoglycaemia and diabetic ketoacidosis (DKA) are also five times higher among young adults than older adults with T1DM [3]. In a metropolitan hospital in Sydney, Australia, 38 % of young adults with T1DM experienced multiple DKA episodes, and 13 % experienced severe hypoglycaemic events [4,5]. Mental health conditions are also prevalent in this cohort, affecting 58 % of those with T1DM and 55 % with T2DM [4,5] 4,5 aligning with international findings where up to 63 % of patients report symptoms of depression and 32 % experience anxiety [6]. Mortality is increased in association with acute glycaemic events, accidents and suicide [7,8]. For young women with diabetes that is not optimally managed, pregnancies can also result in increased risk of adverse pregnancy outcomes and congenital abnormalities in the offspring [9,10].

The transition from youth to adulthood brings unique challenges for young people with diabetes and is often associated with increased risk-taking behaviours, changes in living and employment situations, and heightened financial pressures [11]. This period is also associated with clinic non-attendance, insulin omission, and social isolation. Disordered eating is reported in up to 50 % of young adults with diabetes [12,13], contributing to impaired glucose management. Additionally, 80 % of Australian young adults (19–24 years) do not meet the HbA1c target of 7.0 % [14], with mean HbA1c entry of 10.2 % recorded among young adults with mental health conditions [4]. Diabetes and psychological Interventions addressing risk factors for morbidity and premature mortality have demonstrated improved outcomes in people with diabetes [15,16], however evidence of their effectiveness in young adults remains limited. Given that most young adults are proficient at using smartphones [17], digital app-based health support presents a practical solution to deliver scalable interventions, providing a conduit to maintain mental health level below the threshold for clinical intervention. Perx Health, an Australian company, has developed clinically validated health apps for chronic conditions including T2DM [[18], [19], [20]] which have also been used in cohorts of young adults [20], and has now configured an app to improve mental health and self-management of young adults with diabetes.

The Apps and Peer support for a Healthy future and Living Well with Diabetes (APHLID-M) study will test whether this “app” can feasibly improve distress, well-being and physical health (e.g. glycaemia), while reducing health care costs among young adults aged 16–30 years with diabetes.

## 2 Research question

The APHLID-M study will investigate whether a mobile phone “app” - for young people with diabetes and a mental health condition, improves psychological distress, well-being and physical health (e.g., glycaemia), and reduces health care costs and burden compared with controls receiving usual care. A parallel nested exploratory study among those without a mental health condition using the same methodology will also be included to assess whether the app is of benefit to this group.

## 3 Methods and analysis

### 3.1 Primary and secondary outcomes

The primary outcome, psychological distress, will be measured using the Kessler Psychological Distress Scale (K10). Table 1 provides a summary of the primary and secondary outcomes. A parallel health economic evaluation will also be performed.

**Table 1:** Primary and secondary outcomes, along with their measurement timepoints, for both the intervention and control groups in the Apps and Peer Support for a Healthy Future and Living well with Diabetes (APHLID-M) study.

Outcome Type	Outcome Variable	Measurement/collection timepoint
Primary	Psychological distress assessed using the K10 questionnaire [21]	Baseline and 6 months post baseline
Secondary	HbA1c determined by an accredited laboratory or point of care testing on blood samples collected from participants	
Secondary	Diabetes-related distress measured using the PAID questionnaire [22]	
Secondary	Weight in kg will be measured using digital scale	
Secondary∗	Total number of hospital and/or emergency department admissions	Baseline (during the six month period prior to commencing the trial) and over the six month duration of the trial
Secondary∗	Total number of hospital and/or emergency department admissions due to diabetic ketoacidosis/hyperglycaemia	
Secondary∗	Total number of hospital and/or emergency department admissions due to hypoglycaemia	

K10; Kessler Psychological Distress Scale, PAID; Problem Areas in Diabetes, ∗ assessed using electronic medical records.

### 3.2 Study design

The APHLID-M study includes two multi-site randomised controlled trials (RCTs) in which young adults with diabetes identified as having a mental health condition (MHC) are randomised to receive the app containing diabetes and mental health resources (intervention) or usual care (control group). The study also includes a secondary parallel RCT where participants identified as not having a mental health condition (No-MHC) will be randomised to receive the same app (intervention) or usual care (control). The study was prospectively registered as two separate trials with the Australia New Zealand Clinical Trials Registry: Apps and Peer Support for Young 10.13039/100019769People aged 16–30 years living with Diabetes and Mental Health Conditions (ACTRN12623000734662) and Apps and Peer Support for Young People aged 16–30 years living with Diabetes but without identified Mental Health Conditions (ACTRN12623000733673).

### 3.3 Study phases

#### 3.3.1 Phase 1: app configuration and peer support groups

An app configuration working group, comprising endocrinologists, credentialled diabetes educators, mental health professionals (including psychiatrists and psychologists), academics, consumers, and patient group representatives was established. A list of diabetes and mental health resources, along with website links, was identified and agreed upon by the working group through a Delphi process. The app was configured onto the Perx Health platform (https://www.perxhealth.com). The Perx Health app is based on behaviour change principles including Self-Determination Theory (SDT). It incorporates elements from behavioural science and gamification to improve patient engagement and adherence to health management routines (Supplementary Material) [19]. The modified Perx app (intervention) was organised into two Health Hubs: one for participants with T1DM (Supplementary Material) and another for those with T2DM. For both Health Hubs (T1DM and T2DM) the diabetes resources were made available as hyperlinks under the headings identified in the Delphi process (Table 2). People with lived experience of diabetes (T1DM and T2DM) were invited to review the adapted app, provide feedback, and assess its quality using a validated questionnaire (User Version of the Mobile Application Rating Scale) [23]. Feedback was also obtained from local diabetes clinic psychologists.

**Table 2:** Subject headings under which diabetes and mental health resources will be available in the configured app for the APHLID-M study.

Diabetes Resources	Mental Health Resources
Glucose Management	Mindfulness apps and Online Resources
Insulin Pumps (T1DM only)	Anxiety
Injections	Depression
Sick Days	Stress
Food	Eating Disorders
Contraception and Pre-pregnancy	General Mental Health
Living with Diabetes	

T1DM - Type 1 diabetes mellitus.

For the trial, all resources will be accessible in the Health Hubs for participants to use ad libitum over six months. Additionally, participants will receive in-app communications including SMS messages, alerts and tasks, approximately every four to five days directing them to specific resources within the Health Hub.

#### 3.3.2 Phase 2: APHLID-M randomised controlled trials

##### 3.3.2.1 Study sites and timetable

The APHLID-M study will be conducted across eight Australian sites with separate clinics for young adults in New South Wales (Campbelltown Hospital (lead), Liverpool Hospital, Bankstown-Lidcombe Hospital, St George Hospital, Prince of Wales Hospital, St Vincent's Hospital, Sydney Children's Hospital Westmead) and Victoria (Goulburn Valley Health).

• Commence recruitment: July 2023.
• Complete recruitment: April 2024.
• Complete data collection: October 2024

##### 3.3.2.2 Eligibility criteria and screening

Adolescents and young adults with T1DM, T2DM, or rare forms of diabetes attending diabetes outpatient clinics at any of the eight participating sites will be screened for potential enrolment in the study. Eligible patients will be: aged 16–30 years old; own a smartphone (Apple or Android); able to provide written informed consent. Patients unable to provide written or online informed consent, do not have a smartphone, or who are currently pregnant, or who do not speak English (app only available in English), will be ineligible to participate in the study. All consenting participants will receive a physical activity wearable tracker. No additional vouchers or monetary incentives will be provided.

##### 3.3.2.3 Recruitment

Participants will be recruited by a Research Assistant at outpatient diabetes clinics, including transition, young adult, paediatric, and other clinics, either when attending the clinic or if on the clinic register. The Research Assistant will meet potential participants face to face or contact eligible patients via phone/email to invite them to participate.

##### 3.3.2.4 Screening and allocation of participants to study groups

**Fig. 1:**
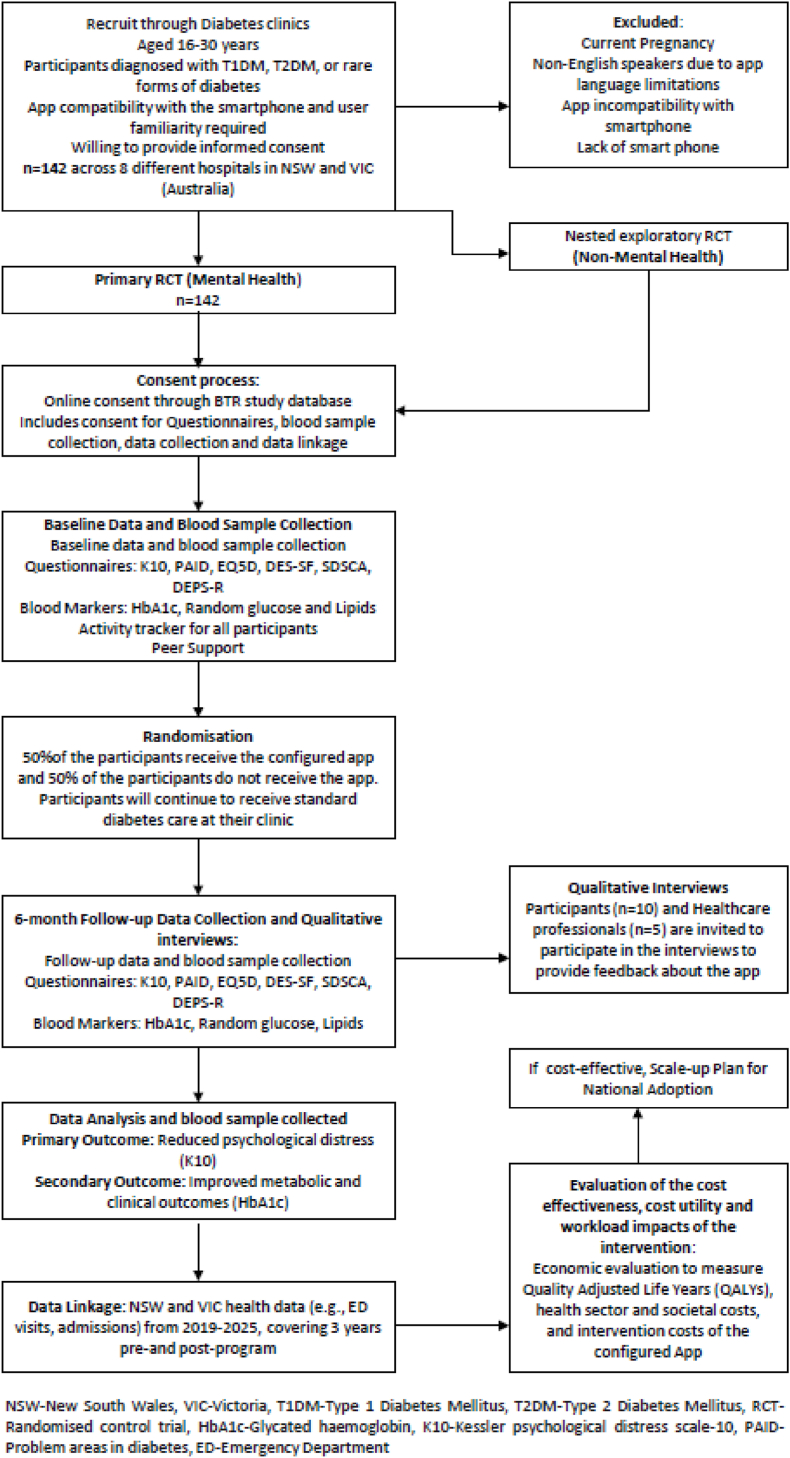
Apps and Peer support for a Healthy future and Living well with Diabetes (APHLID-M) study design.

Participants will be screened using two validated screening questionnaires: the Kessler Psychological Distress Scale (K10) [21] and the Problem Areas in Diabetes (PAID) scale [22]. The K10 is a validated and widely used self-reported psychological screening tool consisting of 10 questions about emotional states, each with a five-point Likert response scale ranging from 1 (none of the time) to 5 (all of the time). K10 total scores range from 10 to 50 with higher scores indicating greater levels of psychological distress [21]. Participants returning a K10 score ≥20 will be classified as having an MHC. The PAID questionnaire measures diabetes-related emotional distress and is widely used in clinical and research settings [22]. The PAID questionnaire includes 20 items covering emotional challenges experienced by people with diabetes, including worries about managing the condition, fears of potential complications, and feelings of frustration or burnout. Participants returning PAID scores ≥40 will be classified as having an MHC [22]. Participants who self-report a previously diagnosed mental health history or are currently receiving care for a mental health condition from a psychologist will also be classified as having an MHC. Participants identified as having major distress (K10 ≥ 40, PAID ≥20) will be considered for referral to the site psychologist (if available) or their general practitioner for management independent of their allocated group but will not be excluded from the study. (Fig. 1). Results from the screening questionnaires will be used to allocate participants into two studies: those with a mental health condition (MHC; Primary RCT) and those without a mental health condition (No-MHC; Nested exploratory RCT).

##### 3.3.2.5 Randomisation and blinding

Randomisation to either “app” or “no app” within each study (MHC and No MHC) will be allocated by the database randomiser using randomisation by minimisation stratified within site. Allocation will not be concealed from participants or healthcare workers, however research staff collecting data will be blinded at baseline and follow-up. Participants assigned to the intervention arms will be onboarded to the Health Hub within the Perx app appropriate to their diabetes type. Participants will be invited to onboard via email and text message notifications (SMS) from Perx Health, which will include instructions on how to download the app and sign in on their smartphones. Research Assistants collecting data and the study statistician will be blinded to a participant's randomisation status. Allocation to the app will not be concealed from participants or healthcare workers.

##### 3.3.2.6 Intervention

The intervention delivered to the MHC and No MHC studies will be identical. All participants will receive standard diabetes care through their regular clinic. This includes education sessions, a food diary, self-management practices such as blood glucose monitoring, healthy eating, regular clinic attendance with review of blood glucose monitoring and weight measurements conducted either by the educator and/or endocrinologist. Participants will receive a physical activity tracker and have access to Peer Support facilitated by people with lived experience of diabetes. Availability of Peer Support is considered part of best practice standard care [24] and will be available at the outpatient clinics of the study sites included in this trial. It is already available at some sites and will be established at sites without existing Peer Support during the trial set-up phase. Peer support can be provided digitally (e.g., ad hoc Facebook communication, Zoom meetings) or through one-on-one or group face-to-face meetings, with the topic focus, frequency and duration determined by the peers or peer support facilitators.

##### 3.3.2.7 Collection of participant data

Quantitative data collected from participants in both RCTs (primary; MCH and nested exploratory RCT; No MHC) at baseline and six months post baseline is described in Table 3.

**Table 3:** Overview of variables and data collected for the APHLID-M primary RCT (MHC) and parallel nested exploratory RCT (No-MHC). Data will be collected from young adults aged 16–30 years with Type 1 diabetes, Type 2 diabetes, or rare forms of diabetes attending specialist diabetes services across eight hospitals.

Clinical Outcomes (measured at baseline and after 6 months)	
Psychological distress	K10 [21]; Diabetes distress [22]
Quantitative data to assess metabolic management	HbA1c
	If testing/using Continuous Glucose Monitoring Sensor/Flash Glucose Monitoring System: Time in/above/below range, glucose variability; mean glucose; blood pressure; lipids; BMI; weight, height (baseline only); CVD risk-adiponectin
Hospitalisation	Number of emergency department or hospital admissions or ambulance treatment and released in the last 3 years (i) total admissions ii) diabetic ketoacidosis (DKA)/hyperglycaemia (iii) hypoglycaemia
Patient engagement	Missed outpatient clinic appointments in the past 6 months; provision of self-monitoring data;
	Medication adherence; sick day planning; hypo treatment; Contraception use; needle/set changes; app use.
Psychosocial and behavioural outcomes	The Diabetes Eating Problem Survey-Revised [DEPS-R) [25] Self-efficacy
Self-management	Summary of Diabetes Self-Care Activities (SDSCA) [26,27] Physical activity tracker time spent in low, moderate, and vigorous activity and sedentary behaviour, time spent sleeping and quality of sleep.
Patient reported outcomes	Diabetes Empowerment Scale – Short Form (DES-SF) [28]
Sample collection and storage	Samples will be taken for the measurement of total/high-molecular-weight adiponectin using a quantitative monoclonal sandwich ELISA and stored for use in future studies of diabetes and its risk factors and complications.
Cost-Utility	EQ5D-5L [29,30]; Hospital service utilisation including telephone and online occasions of service; Acute glycaemic hospitalisation/emergency department visits; Economic evaluation includes Institute for Clinical and Economic Review.
Other: includes qualitative research	Consumer and healthcare professional perspectives on the intervention and its impacts on patient, provider, and health-system implementation

HbA1c-glycated haemoglobin, BMI – body mass index, CVD-cardiovascular disease, ED-emergency department, ELISA – enzyme linked immunosorbent assay, PA-physical activity, MHC – mental health condition, No-MHC – No mental health condition.

To gain insight into participants' experiences and perceptions of using the app and its impact on diabetes management, participants randomised to the intervention from both studies will be invited to participate in focus groups or one on one interviews (online or face to face) as per the participants preference/availability. Additionally, and separately, healthcare staff from each site will be invited to participate in interviews/focus groups, to provide feedback on perceived feasibility of wider rollout of the intervention, and its long term sustainability at their clinics.

All participants (MHC and No MHC) will be invited to provide consent for linkage of their study data to the health system administrative data. Data requested from participants in the NSW sites are held in the Master Linkage Key (MLK) at the Centre for Health Record Linkage (CHeReL) and will include: Ambulance Data Collections; Emergency Department Data Collection (EDDC); NSW Admitted Patient.

Data Collection (APDC); NSW Mental Health Ambulatory Data Collection; NSW Non-admitted Patient Data Collection (NAP); and cause of death and register of death. The same data will be requested for the Victorian site (Goulburn Valley Health), from the Centre for Victorian Data Linkage. The data will be requested for the period 2019 to 2025 and to enable 3 years pre-trial and 3 years post-trial comparisons.

##### 3.3.2.8 Blood sample collection and storage

Non fasting blood samples for HbA1c, plasma glucose (in Flouride-EDTA tube), serum lipids and adiponectin will be collected from hospital phlebotomy services or by qualified Research Nurses at each site. Following collection, samples will be processed within 30 min and stored at −80 °C until analysis.

#### 3.3.3 Phase 3: evaluation of the cost effectiveness, cost utility and workload impacts of the intervention

The primary outcome for economic evaluation is the Quality Adjusted Life Years (QALYs), as measured by EQ5D-5L that will be completed at baseline and at the follow-up assessment for both groups (primary RCT and nested observational RCT).

The Australian health sector and societal perspectives will be used for the analyses to reflect the different decision makers and contexts and per the current guidelines for reference cases in economic evaluations. The health sector costs include those paid by the government of primary and acute care, medication costs, and ambulatory costs as well as the out-of-pocket costs of health care resources paid by the participants during the trial period. Societal costs comprise the health sector costs in addition to the costs of patient transportation (costs per km travelled for visits to health care providers), food and effects on productivity (labour market earning loss of paid work and unpaid lost productivity due to illness). In addition, costs to carers and/or support person(s) will be determined including direct and indirect costs.

The intervention costs of using the adapted Perx app will include training and maintenance costs specific for the RCTs. Costs associated with utilisation of psychologists will be included in the health economic analysis of the primary RCT, and may be included in the exploratory subgroup analyses of the nested observational RCT if a difference is found.

#### 3.3.4 Phase 4: scale up plan for intervention

A distribution plan will be developed through health services, diabetes consumer organisations, mental health consumer organisations, and health professional organisations (e.g. Australian Diabetes Educators Association which would then reach those in the private as well as the public sector, Pharmaceutical Society of Australia (PSA) and Pharmaceutical Guild of Australia (PGA) i.e. through pharmacists).

### 3.4 Statistical analysis

For the primary outcome variable (K10), a linear mixed effects regression model will be used to show any change, with participant identification number and study site as random effects. Age, gender, ethnicity, socio-economic status, type of diabetes, time and intervention group will be included as fixed effects. A significant interaction between intervention group and time (Baseline vs Follow Up) would indicate that the average change in K10 was significantly different between intervention and control groups [31]. This is the main test of the study and will be subjected to a significance level of α = 0.05. Level of significance will also be indicated using p-values. The effect sizes will be presented as the difference between the average decrease in K10 between intervention groups.

A linear mixed effects regression model will also be used for the secondary outcome variables including a change in; (1) HbA1c (2) PAID and (3) weight in kilograms. To account for multiple testing, a false discovery rate method such as the Benjamini-Hochberg procedure [32], will be adopted for the secondary outcome variables. The effect sizes will be presented as the difference between the average change in outcome between intervention groups. Results will be visualised using a forest plots. For all linear mixed effects models, model diagnostics will be conducted, including, but not limited to (a) examining the residuals for departures from normality, (b) collinearity between covariates using the variation inflation factor and (c) examination of outliers to see if they have too much influence on the results of the analysis. Consequential departures from normality and/or highly influential outliers will be handled using bootstrapping or through transformation of the outcome variable.

A generalised linear mixed effects regression model with a Poisson error model [33] will also be used for the secondary outcome variables; (4) the total number of hospital and/or emergency department admissions and (5) the total number of hospital and/or emergency department admissions due to diabetic keto-acidosis/hyperglycaemia. The models will be examined for over or under dispersion and a more appropriate error model will be adopted if necessary.

Analyses will be adjusted for the following confounders: age, gender, ethnicity, socio-economic status (measured using postcode and Socio-Economic Indexes for Areas tool (SEIFA: Australian Bureau of Statistics), study site, and type of diabetes. The methods adopted will be the same for the primary (MHC) and nested exploratory (No-MHC) RCTs. If necessary, multiple imputation by chained equations [34] will be used to provide intention-to-treat results.

Interviews with participants and healthcare professionals will be conducted utilising the Reach, Effectiveness, Adoption, Implementation, and Maintenance (RE-AIM) framework to evaluate community and clinical impact [35]. The interviews will assess project engagement and implementation, effects of the app, and feedback and recommendations on app content and design. Interviews will be transcribed, coded to identify themes and sub-themes in NVivo and undergo reflexive thematic analysis to present key findings. Reflexive thematic analysis was chosen due to its high recommendation by leading qualitative researchers in the context of healthcare interventions with psychological impact and social outcomes [36]. Briefly, a 6-step process to analysis will involve: 1) data cleaning and familiarisation; 2) coding, where codes relate to features of the data that has been generated; 3) theme development through organisation of codes into broader themes across the dataset; 4) theme review and refinement; 5) theme naming and defining; and 6) reporting of findings.

#### 3.4.1 Missing values

Electronic data entry and follow up procedures will be used to minimise missing data. Attrition will be minimised through participant follow up including multiple attempts to re-contact though clinic visits, telephone, email and text. Missing values will be assessed to determine if they are random. If the missing data are found to be non-random, an intention-to-treat analysis will be conducted, and multiple imputation methods will be applied.

#### 3.4.2 Sensitivity analysis

A per protocol analysis will be performed and compared with the results of the intention-to-treat analysis. For the per protocol analysis, only those participants considered to have received the Perx app will be included in the analysis. Participants will be considered to have used the Perx app if they have logged into the app and completed at least one task during the intervention period.

#### 3.4.3 Power calculations

Based on data published previously (behavioural intervention K10 change 21.2 ± 9.4 vs baseline 25.7 ± 9.7 Cohen d = 0.5), a sample size of 64 per group in the primary RCT will be required to identify a moderate effect size of 0.5 between intervention and control group, in a *t*-test model with power = 0.8, alpha 0.05. An additional 14 participants will be included to allow for an attrition rate of 10 % (total n = 142). The nested exploratory RCT (No MHC) is considered an exploratory study as it could not be powered.

#### 3.4.4 Data management

A customised web-based data capture system (BTR; Western Sydney University, Australia) will be employed to gather data, which will be stored at the primary analysis and study coordination site located at Western Sydney University, Campbelltown, Australia. Perx will capture app metrics, including enrolment details, app utilisation (e.g., average sessions per day, average time spent in the app per month, utilisation of diabetes and mental health resources), task and medication adherence, participant satisfaction, and content engagement. The time spent using the app, linked to in-app communications is considered an exploratory outcome.

## 5 The role of Perx Health in APHLID-M

The app provider, Perx, are contractors and will receive payment through the grant. Perx will not fund any part of the study or have input into the study design. Perx's role is limited to supplying the platform and managing the configuration of the existing Perx app to include diabetes and mental health resources, identified and agreed upon by the app configuration group. Additionally, Perx will oversee the onboarding of participants assigned to the app group but will not be involved in data analysis or interpretation of study results.

## 6 Dissemination of findings

Results will be disseminated in peer-reviewed publications, at scientific meetings, through collaborations and discussions with stakeholders and policy makers.

## 7 Discussion

This is the first multi-site RCT to evaluate whether a configured health “app” containing diabetes and mental health app improves psychological distress, well-being, and physical health in young adults with diabetes and a MHC. The design allows for a parallel exploratory RCT among those without a MHC.

In this study the K10 will be used as a tool to screen for psychological distress (primary outcome measure), as it offers a broad, general assessment of mental health [21]. This decision was based on the high levels of psychological distress and other mental health challenges commonly experienced by young people with diabetes [37,38]. Additionally, when diabetes co-occurs with mental health disorders, there is a cumulative effect on both mental and physical health, leading to a reduced quality of life [39,40]. The K10, widely used internationally, enables comparison of distress levels across diverse clinical conditions (Kessler et al., 2003) providing a more comprehensive measure of MHC beyond diabetes. In contrast, the PAID scale specifically measures diabetes-related distress [22], which, while valuable, limits the scope of the distress captured to diabetes-related stressors only. Since the APHLID-M study aims to address mental health needs of young people with diabetes, HbA1c was designated as a secondary outcome, with its relationship to psychological distress to be explored in the analysis.

The APHLID-M study design integrates peer support and the use of activity trackers for all participants. Peer support, is a standard part of care across all study sites and has been shown to be beneficial for managing mental health conditions in people with diabetes [41] and improve behavioural outcomes (e.g., medication adherence), psychosocial factors (e.g., diabetes distress), and health measures (e.g., blood glucose levels and HbA1c) in people with diabetes [42]. Activity trackers are provided to participants to incentivise participation, promote engagement and adherence to physical activity and health goals throughout the study. This approach addresses both psychological and physical aspects of diabetes, contributing to better overall well-being and clinical outcomes.

While it is anticipated that most participants recruited from the hospital clinics will have T1DM, people with T2DM or rare forms of diabetes will also be included in this study, and as such the app was correspondingly configured to contain two Health Hubs. The inclusion of people with T2DM is important as they also experience significant mental health challenges [43] and are at elevated risk for long-term diabetes related complications [44]. This approach will enable the evaluation of the intervention's effectiveness across different diabetes phenotypes and aligns with the study's objectives to improve both diabetes and mental health outcomes. It also ensures the intervention's applicability across different diabetes types. Additionally, recruiting all young adults and then allocating them to study groups based on mental health status helps reduce the stigma often associated with mental health in diabetes [45].

A key strength of this study is its RCT design, with blinding of the research team, which ensures a high level of scientific rigor by minimising bias and allowing for a clear evaluation of the apps impact on mental health and diabetes outcomes. Another key strength of the study design is the inclusion of participants aged 16–30 years, capturing young patients across a wide age range enabling a comprehensive analysis of how the app intervention impacts mental health and diabetes outcomes across the 16 to 30-year age range. Including participants from both urban and rural areas enhances the study's generalisability, ensuring its findings are applicable across different geographic and socio-economic settings. The inclusion of a health economic analysis and qualitative evaluation of participants experience using the app further enhances the value of this study.

The sample size of the primary RCT (n = 142) was calculated allowing for a 10 % loss to follow-up. However greater loss to follow-up due to poor participant attendance at follow-up assessments could impact the ability to detect a moderate effect size of 0.5 in K10 score between intervention and control group. Given that the participants are attending clinical services and the trial duration short, it is hoped that follow-up targets are achieved. Nonetheless participant responsiveness is uncertain. To minimise loss to follow-up, participants will be contacted via text messages, email, and clinic or study phones as necessary. Additionally, the short follow-up period limits insights into the sustainability of the effects if observed. Due to the smaller sample size expected in the nested exploratory No-MHC arm, no independent power calculations were performed. Despite separate registrations, both groups will receive identical interventions and follow the same study procedures. Publishing a single protocol paper ensures clarity, avoids unnecessary duplication, and maintains consistency in reporting across both study arms. While a formal pilot study specific to youth with diabetes has not been conducted, the platform has been used among young people with cystic fibrosis, some of whom have cystic fibrosis related diabetes. Nevertheless, a post-trial qualitative analysis will be undertaken with participants randomised to the app, to assess user experience and gather suggestions for improvements.

In conclusion, the APHLID-M study represents the first multi-site RCT to assess the effectiveness of an app containing diabetes and mental health resources in improving mental health, well-being, and physical health in young adults with diabetes with and without MHC. The study incorporates peer support and activity trackers to promote participant engagement and behaviour change and improve health outcomes. Data from activity trackers will be collected where available. With its diverse cohort of paediatric and adult participants from both urban and rural settings, the study aims to provide valuable insights into how the intervention can be scaled and its potential impact on reducing healthcare costs. While challenges related to follow-up rates and sustainability exist, the study's design ensures robust evaluation of the intervention's effect on mental health and diabetes outcomes across different populations and settings.

## CRediT authorship contribution statement

**K.O. Mathews:** Writing – original draft, Methodology. **F. MacMillan:** Writing – review & editing, Methodology, Funding acquisition. **V. Wong:** Writing – review & editing, Funding acquisition. **M. Craig:** Writing – review & editing, Funding acquisition. **J.R. Greenfield:** Writing – review & editing, Funding acquisition. **R. Hicks:** Writing – review & editing, Methodology, Funding acquisition. **T. Jones:** Writing – review & editing, Funding acquisition. **A. Poynten:** Writing – review & editing, Funding acquisition. **T. Wong:** Writing – review & editing. **M. Reyes:** Writing – review & editing. **K. Tannous:** Writing – review & editing, Methodology, Funding acquisition. **C. Wilson:** Writing – review & editing, Methodology. **P. Hay:** Writing – review & editing, Methodology. **S. Abdo:** Writing – review & editing. **M.K. Piya:** Writing – review & editing, Funding acquisition. **J. Lai:** Writing – review & editing. **M. Venigalla:** Writing – review & editing. **R. Thomson:** Writing – review & editing, Methodology. **D. Simmons:** Writing – review & editing, Supervision, Project administration, Methodology, Funding acquisition, Conceptualization.

## 4 Ethics approval

The study was approved by the South Western Sydney Local Health District Human Research Ethics Committee (reference PID:2022/02828, 2022/ETH02515).

## Ethics approval

Ethics approval: South Western Sydney Local Health District Human Research and Ethics Office (reference PID:2022/02828, 2022/ETH02515).

## Trial registration

Australian New Zealand Clinical Trials Registry, ACTRN12623000734662 (Primary RCT; mental health condition) and ACTRN12623000733673 (nested exploratory RCT; No mental health condition)

## Funding statement

This trial is funded by a grant from the Australian Government Department of Health and Aged Care (MTP Connect-Targeted Translation Research Accelerator (TTRA) for Diabetes and Cardiovascular Disease. In-kind contributions were provided by the Sydney Partnership for Health, Education, Research and Enterprise (SPHERE), the Butterfly Foundation, and Headspace.

## Declaration of competing interest

The authors declare that they have no known competing financial interests or personal relationships that could have appeared to influence the work reported in this paper.
