# Supplementary material for: Study protocol: Apps and peer support for a healthy future and living well with diabetes (APHLID-M)
Source: Contemp Clin Trials Commun. 2025 Apr 14;45:101484. doi: 10.1016/j.conctc.2025.101484 (PMC12052685; doi:10.1016/j.conctc.2025.101484)
Supplement: Multimedia component 1 [file mmc1.docx]

**Supplementary Material: Apps and Peer support for a Healthy future and Living with Diabetes (APHLID-M)**


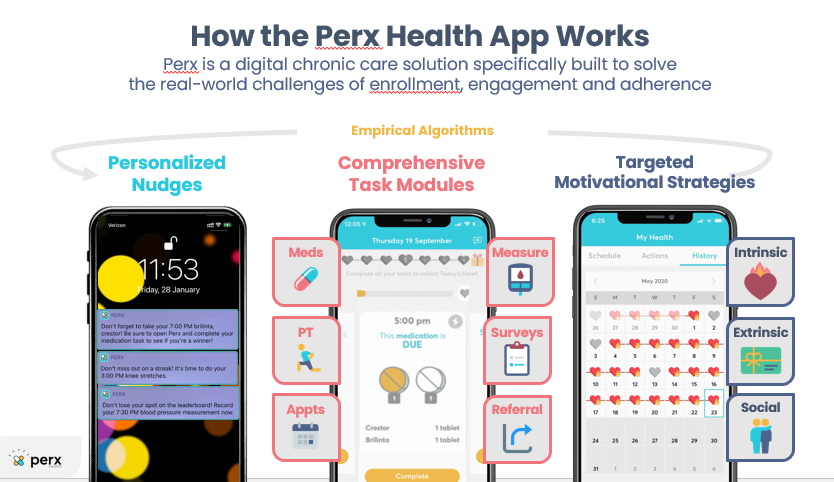


**Figure 1.** The Perx app simplifies health management by consolidating daily tasks such as taking medications, attending appointments, and performing physiotherapy exercises—into one easy-to-follow schedule. It employs personalised, engaging in-app nudges to remind users when tasks are due and motivates them through intrinsic rewards like daily streaks, as well as extrinsic incentives such as gift vouchers upon task completion. Additionally, the app enables users to track their progress using clinical measurements and condition-specific surveys while also providing access to tailored content relevant to their specific health conditions


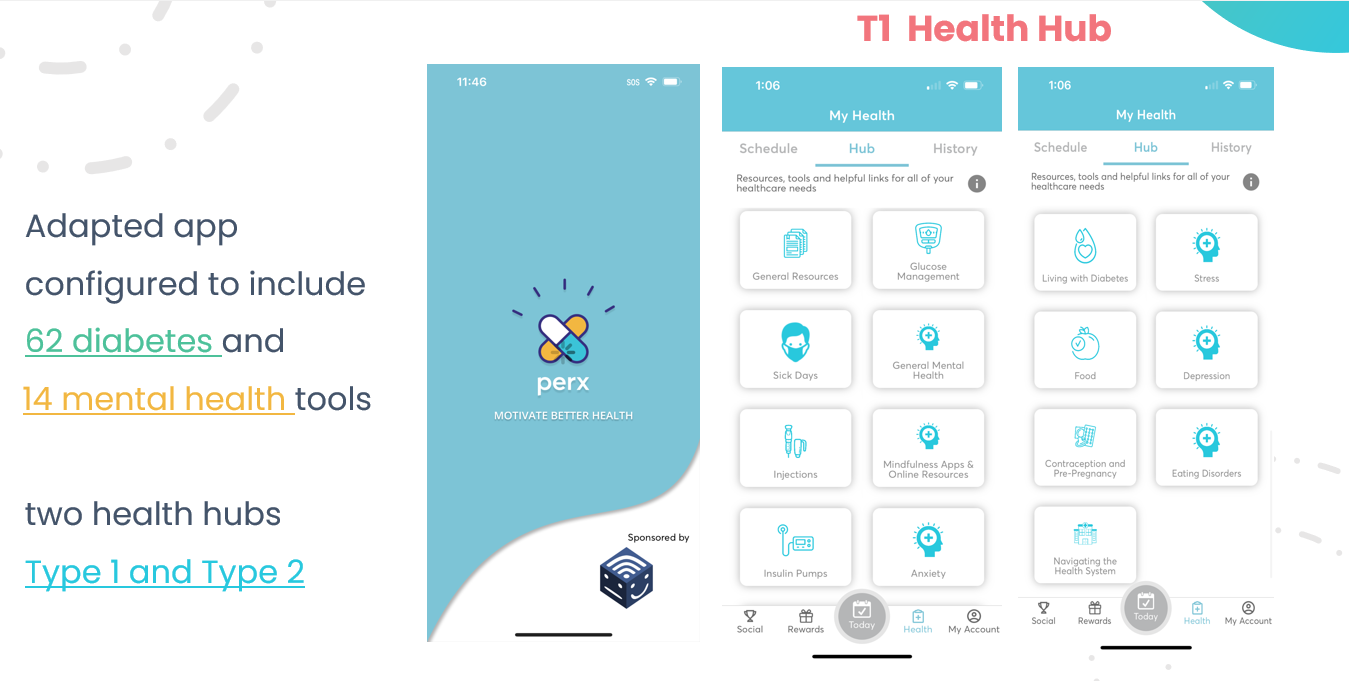


**Figure 2.** The final version of the adapted app to be used in the trial incorporates 62 diabetes tools and 14 mental health tools and is configured with two distinct health hubs—one for individuals with Type 1 diabetes and another for those with Type 2 diabetes. Above is an example of the T1 health hub with diabetes resource tiles on the left hand column and mental health resource tiles on the right


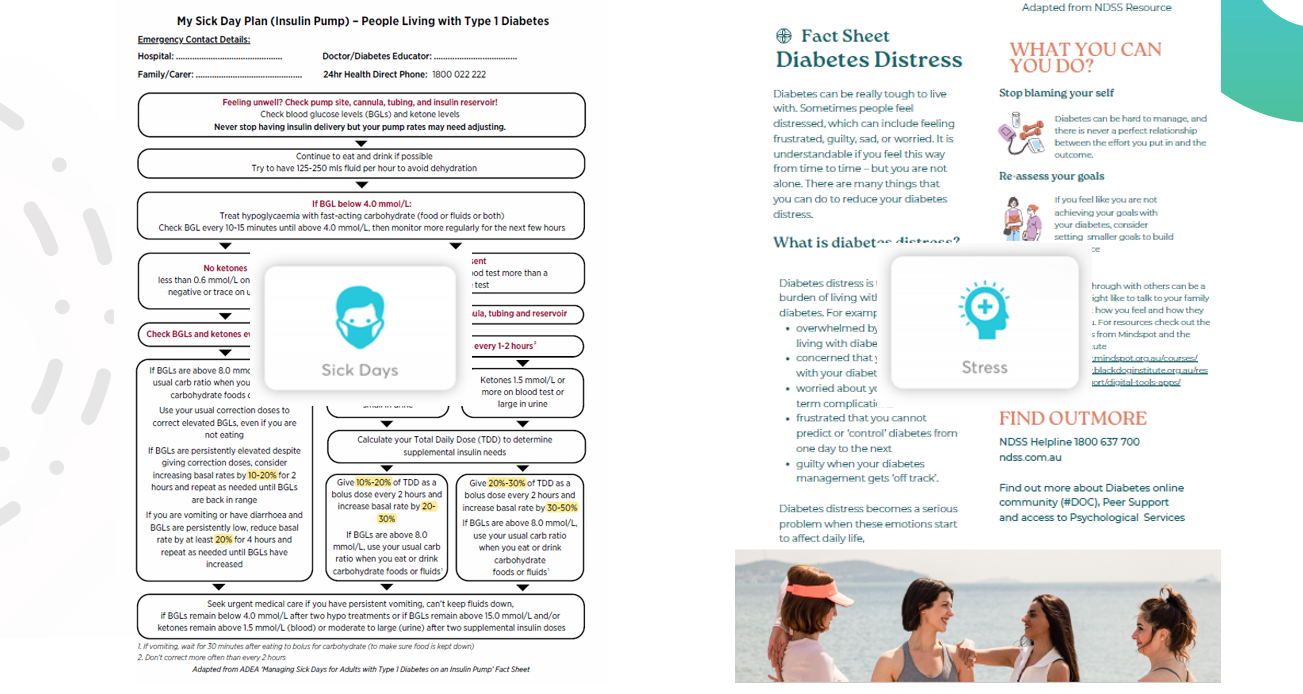


**Figure 3.** The above figure illustrates the screens displayed to participants after they click on the "Sick Days" or "Stress" tiles, as shown in **Figure 2**.
